# Utilizing Artificial Intelligence and Remote Sensing to Detect *Prosopis juliflora* Invasion: Environmental Drivers and Community Insights in Rangelands of Kenya

**DOI:** 10.3390/plants13131868

**Published:** 2024-07-06

**Authors:** Ambica Paliwal, Magdalena Mhelezi, Diba Galgallo, Rupsha Banerjee, Wario Malicha, Anthony Whitbread

**Affiliations:** 1 International Livestock Research Institute, P.O. Box 30709, Nairobi 00100, Kenya; d.galgallo@cgiar.org (D.G.); b.rupsha@cgiar.org (R.B.); w.malicha@cgiar.org (W.M.); 2 International Livestock Research Institute, c/o IITA, Mwenge Coca-Coal Road, Dar es Salam 34441, Tanzania; magdamhelezi99@gmail.com (M.M.); a.whitbread@cgiar.org (A.W.)

**Keywords:** artificial intelligence, machine learning, remote sensing, invasive species, *Prosopis juliflora*, rangelands, environment, Kenya, Africa

## Abstract

The remarkable adaptability and rapid proliferation of *Prosopis juliflora* have led to its invasive status in the rangelands of Kenya, detrimentally impacting native vegetation and biodiversity. Exacerbated by human activities such as overgrazing, deforestation, and land degradation, these conditions make the spread and management of this species a critical ecological concern. This study assesses the effectiveness of artificial intelligence (AI) and remote sensing in monitoring the invasion of *Prosopis juliflora* in Baringo County, Kenya. We investigated the environmental drivers, including weather conditions, land cover, and biophysical attributes, that influence its distinction from native vegetation. By analyzing data on the presence and absence of *Prosopis juliflora*, coupled with datasets on weather, land cover, and elevation, we identified key factors facilitating its detection. Our findings highlight the Decision Tree/Random Forest classifier as the most effective, achieving a 95% accuracy rate in instance classification. Key variables such as the Normalized Difference Vegetation Index (NDVI) for February, precipitation, land cover type, and elevation were significant in the accurate identification of *Prosopis juliflora*. Community insights reveal varied perspectives on the impact of *Prosopis juliflora*, with differing views based on professional experiences with the species. Integrating these technological advancements with local knowledge, this research contributes to developing sustainable management practices tailored to the unique ecological and social challenges posed by this invasive species. Our results highlight the contribution of advanced technologies for environmental management and conservation within rangeland ecosystems.

## 1. Introduction 

The rapid spread of invasive alien plant species poses a significant threat to the biological productivity of native vegetation and communities within the rangelands of Africa leading to land degradation. Introduced in the 1880s, *Prosopis juliflora* was initially welcomed for its potential to provide fodder, shade for livestock, fuelwood, windbreaks for erosion control, and timber [1,2,3]. Yet, its capacity to thrive and proliferate in arid and semi-arid settings has led to its invasive status. The invasion by *Prosopis juliflora* across African drylands and semi-drylands is because of its biological characteristics, favorable environmental conditions, and anthropogenic activities. Remarkably adaptable, it can withstand drought [1], high temperatures, and poor soil quality [4] outcompeting native vegetation and colonizing areas. Known for its rapid growth and extensive seed production, a single *Prosopis juliflora* tree can generate thousands of seeds each year. These seeds are widely dispersed by animals, water, and human actions, aiding in its spread and the establishment of new colonies. Furthermore, human activities such as overgrazing, deforestation, and land degradation exacerbate conditions conducive to its invasion. Overgrazing diminishes competition from native grasses and shrubs, allowing *Prosopis juliflora* to spread unchecked. This invasion significantly impacts biodiversity, agriculture, ecosystem services, and economic development. When monitoring forage cover from space, the presence of this invasive species can misleadingly indicate healthy undergrowth, leading to an overestimation of forage quality and quantity for livestock grazing. This misrepresentation can adversely affect the livelihoods of ranchers and pastoralists who rely on these lands for their sustenance.

Accurate and timely information on the spatial and temporal distribution of invasive species is essential for effective management and control efforts [5]. Such information is vital for understanding the spread and impact of invasive species. Traditional field surveys, while useful, are often time-consuming and require substantial financial resources [6]. Consequently, there has been a significant shift towards utilizing satellite remote sensing to monitor and differentiate invasive species from palatable forage [7]. Satellite imagery provides continuous coverage, enabling a deeper understanding of the phenology of both native vegetation and invasive species [8]. Multispectral images from optical sensors are increasingly employed for mapping invasive species on both local and regional scales, with spatial resolutions of 10 m and above being the most utilized for such purposes [7,9,10]. However, analyzing multifaceted data sources can be labor-intensive. 

To enhance the accuracy, accessibility, and affordability of satellite-based observations, integrating machine learning (ML) algorithms have emerged as a powerful approach for environmental modeling, analysis, and decision-making. ML models are uniquely capable of adapting and improving as they process more data, a feature that is particularly advantageous in dynamic ecosystems like rangelands, where ecological conditions and management challenges can evolve swiftly [11,12,13,14]. Recent studies have begun to explore the use of ML algorithms for invasive species monitoring, showcasing promising results [15,16]. This integration not only streamlines the data analysis process but also leverages the vast amount of data generated by satellites, opening new avenues for understanding and managing the ecological impacts of invasive species.

Effective management of rangelands extends beyond mere monitoring and mapping of invasive species. It critically encompasses understanding community perceptions regarding these species. Studies have shown that stakeholder engagement is crucial for controlling invasion [17]. Opinions on *Prosopis juliflora* vary widely, with some viewing it as a detriment to biodiversity and the environment, while others rely on it for their livelihoods. Bearing these considerations in mind, our study aims to investigate the utility of machine learning algorithms in precisely detecting *Prosopis juliflora* in the rangelands of Kenya using satellite imagery and other environmental variables. Additionally, we also aim to identify and quantify the important explanatory variables that contribute most in detecting *Prosopis juliflora* from the native vegetation, as well as to gauge the local community’s perceptions.

Our study aims to address the following research questions:

1. Which machine learning algorithm is most successful in predictive detection of *Prosopis juliflora*?
2. What are the key variables contributing to distinguish *Prosopis juliflora* presence in arid and semi-arid ecosystems?
3. How does the community perceive *Prosopis juliflora* presence in the rangelands?

This comprehensive approach aims not only to enhance our scientific understanding of *Prosopis juliflora*’s spread and control but also to integrate community insights into more effective and sustainable management practices. 

## 2. Results 

### 2.1. ML Algorithm Comparison 

We conducted an evaluation of three machine learning algorithms—RF, SVM, and NN (Table 1) using overall accuracy, Cohen’s Kappa coefficient and McNemar’s test. This comparison aimed to identify any statistical differences in their predictive capabilities. The Decision Tree/Random Forest classifier emerged as the most accurate, with a remarkable 95% success rate in classification, showcasing its superior ability. With a Kappa coefficient of 0.53, it indicates a substantial level of agreement beyond what chance would predict. The McNemar’s Test *p*-Value, sitting at 0.9, reveals no significant disparity between the Decision Tree/Random Forest models. On the other hand, the Neural Network classifier displayed lower accuracy and a reduced Kappa coefficient, suggesting less agreement beyond chance in comparison to the other models. Nonetheless, with a McNemar’s Test *p*-Value of 0.07, there is an indication of a distinguishable difference in performance relative to the Decision Tree/Random Forest model, although this did not reach statistical significance.

### 2.2. Key Variables Contributing to the Detection of Prosopis juliflora 

**Figure 1:**
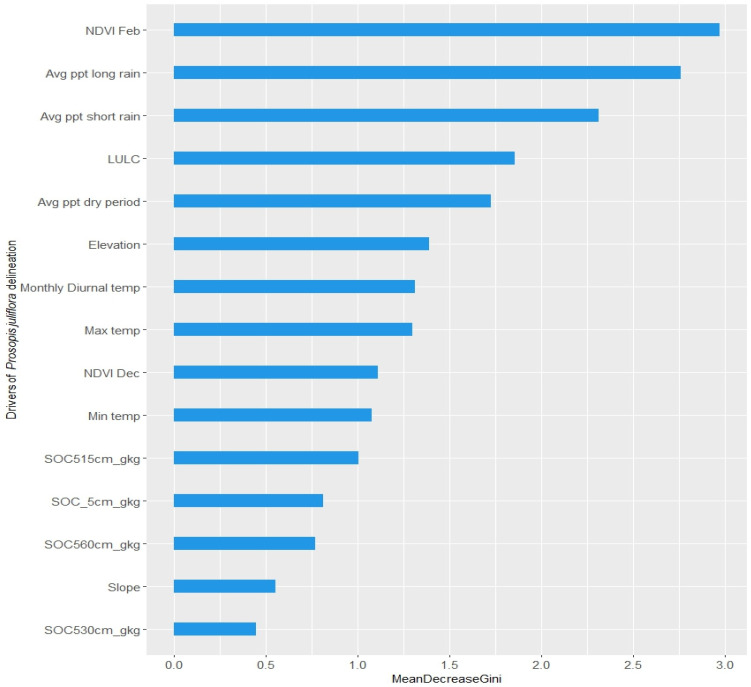
Variable importance plots for the weather, landcover and biophysical factors to explain occurrence of *Prosopis juliflora*. Where NDVI Feb and NDVI Dec is normalized difference vegetation index of February and December month respectively. Avg ppt long rain is mean precipitation for long rain season, Avg ppt short rain is mean precipitation for short rain season, Avg ppt dry period is mean precipitation during the dry period. Temperature variables are Max temp, Min temp and Monthly diurnal temp signifies mean monthly maximum, minimum and diurnal temperatures. SOC_5, SOC515, SOC530 and SOC560 signifies the soil organic carbon at 0–5, 5–15, 15–30 and 30–60 cm depth levels. LULC is land use/landcover class and slope and elevation are biophysical variables used in the study.

**Figure 2:**
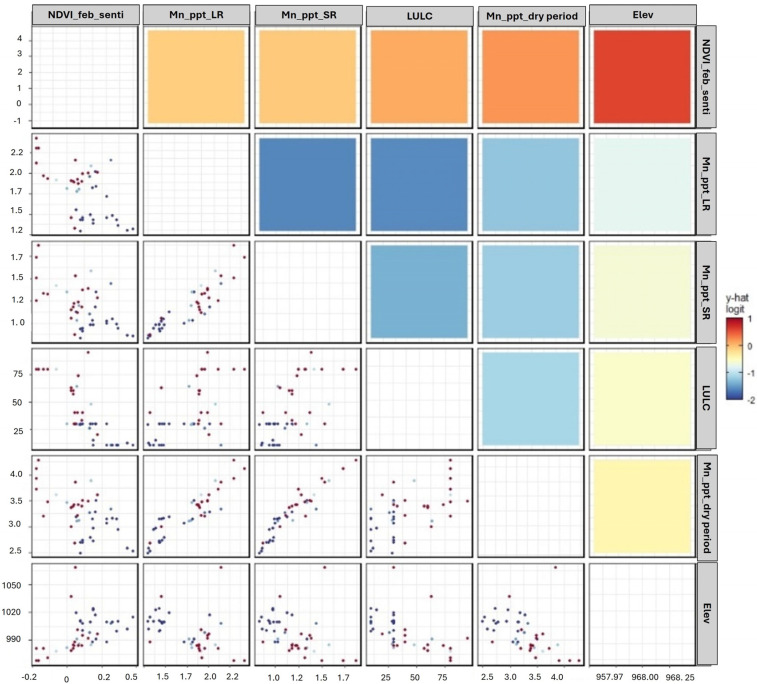
The generalized partial dependence plot (pdp) shows the relationship between variables and the probability of *Prosopis juliflora* presence, with red points indicating presence and blue points an absence. Each row and column correspond to one variable, with diagonal plots providing histogram of the variable marginal distribution. The off-diagonal plots show the pdp relationship between pairs of variables. The color intensity in each plot reflects the magnitude of the predicted logit value, with red indicating higher and blue lower probability.

In analyzing the detection of *Prosopis juliflora* against native vegetation, our study identifies several critical variables across weather, land cover, and biophysical domains that significantly aid in distinguishing this invasive species. Notably, the Normalized Difference Vegetation Index (NDVI) for February emerges as the significant indicator for detecting *Prosopis juliflora*, surpassed only by the aggregated rainfall measurements during both the long and short rainy seasons, as well as the dry period. Furthermore, factors such as land cover classifications, elevation, monthly diurnal temperature variations, and maximum temperatures play a substantial role in the effective identification of *Prosopis juliflora*, the importance of each variable is shown in Figure 1. To further understand the intricate relationship between these variables and the likelihood of *Prosopis juliflora*’s presence, we employed generalized partial dependence plots (Figure 2), derived from the random forest classification model. These plots offer a dual perspective, while diagonal plots showcase the distribution of individual predictors, off-diagonal plots reveal the mutual dependencies between pairs of variables. This is visually represented through a color gradient, where red signifies a higher predicted log-odds of the species’ presence and blue indicates a lower likelihood.

Key factors such as NDVI, precipitation, land use types, and elevation each uniquely influence the model’s predictions. Elevation, for example, demonstrates a profound effect through its extensive color spectrum within the plots. A notable observation is the interaction between February’s NDVI and the average precipitation during the long rainy season, highlighting how variations in these predictors correlate with changes in *Prosopis juliflora*’s presence probability. This approach allows us to not only comprehend the singular impact of each environmental variable on the presence of invasive species but also to uncover the multifaceted and nonlinear interactions between these factors, thereby providing deeper insights into their collective influence on *Prosopis juliflora* detection.

### 2.3. Community Perceptions

**Figure 3:**
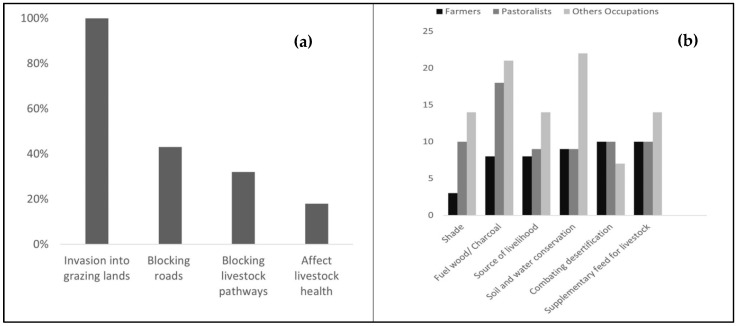
Community perceptions of *Prosopis juliflora*’s impact. (**a**) shows the percentage of households reporting negative effects, including grazing land invasion and blocked pathways. (**b**) details perceived benefits by occupation, highlighting differences among farmers, pastoralists, and other occupations.

Our recent survey has uncovered a heightened awareness among the community regarding the pervasive and detrimental impact of *Prosopis juliflora*. This invasive species has been steadily encroaching upon grazing territories, significantly impairing the health of livestock and consequently the livelihoods that depend on them. We found a unanimous observation among surveyed households, who have experienced a pronounced adverse effect on rangelands for livestock grazing due to *Prosopis juliflora* (Figure 3a). Further data indicated that 32% of the households were contending with impeded grazing routes, while 43% were facing obstructions along access roads crucial for human transit. While the negative aspects were clear, the survey also captured a dichotomy in perception. A segment of the community acknowledged the plant’s beneficial roles, highlighting its utility in providing shade and fuelwood (Figure 3b). It was also credited with contributions to soil and water conservation efforts and helps against desertification. To delve into the nuances of these perceptions, we sorted the responses by various occupational groups, such as farmers, pastoralists, and other occupations, observing the differences in their respective experiences with *Prosopis juliflora*. Pastoralists use it as a feed supplement as an alternative source of protein. Our findings reveal that male respondents were more likely to point out the benefits, doing so at a rate four times higher than that of female respondents. This significant disparity in the recognition of *Prosopis juliflora*’s positive contributions between genders underscored the complexity of community perspectives and the varied impact of this species on different segments of the population.

## 3. Materials and Methods

### 3.1. Study Area

**Figure 4:**
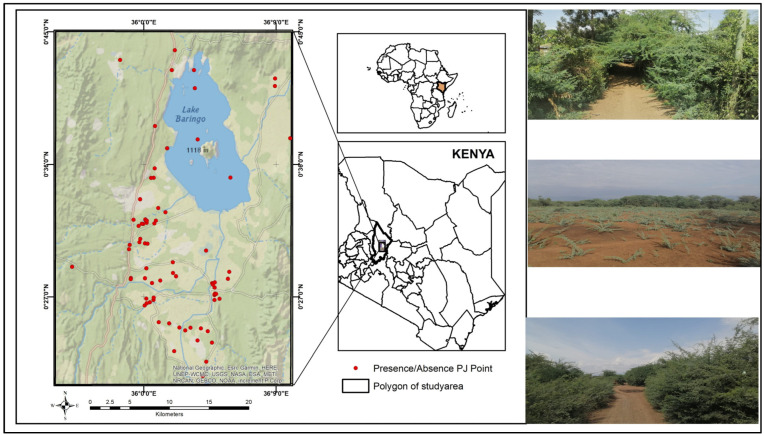
A map depicting the study area in Baringo County, Kenya, with red dots marking the occurrence points of *Prosopis juliflora* (PJ) also features pictures of PJ taken from Baringo County.

We conducted our study in Baringo county, Kenya (0°40′0″ N, 36°0′0″ E) (Figure 4) which is home to Lake Baringo. Annually, temperature variations are observed within a range of 10 °C to 35 °C. The precipitation levels span from 1000 to 1500 mm in the elevated regions and 300 to 700 mm in the lower regions, exhibiting a dual-peak pattern in April and November [18]. The current vegetation predominantly comprises a blend of native and foreign woody species, extending from Vachellia-led deciduous shrubland at the valley’s base to perennial woodlands in the elevated areas [19]. In the lowland plains adjacent to the central-eastern and central-western parts of Lake Baringo and extending southward to the northern extremity of Lake Bogoria, *Prosopis juliflora* is the prevailing species [18,19]. In the past, the flat lowland terrains of Baringo County were characterized by a mixed landscape of grasslands and shrublands [20]. Between 1982 and 1983, the introduction of Prosopis was carried out via the Fuelwood Afforestation Extension Project [21]. This initiative aimed to engage community members in afforestation efforts to address challenges like the scarcity of firewood and advancing desertification [21]. 

### 3.2. Sampling Design, Datasets and Analysis

Field Data Collection: In August 2023, we gathered geo-coordinates to identify where *Prosopis juliflora* was present or absent within the study area, totaling 80 data points on presence and absence of species. We were accompanied by local people with indigenous knowledge and taxonomists to identify the locations in Baringo county where the presence of *Prosopis juliflora* was significantly visible and geo-coordinates were recorded for each such location. We also recorded a geo-coordinate at the point where no trace of *Prosopis juliflora* was found, for instance crop fields, forest and water bodies. Additionally, we surveyed the local population to gauge their perceptions of *Prosopis juliflora*. By employing questionnaires during interviews with key informants, we were able to collect primary data aimed at understanding community awareness and attitudes towards this species. Specifically, we asked questions on where the species is found, has the density of species is increasing or decreasing in last five years, what are the benefits and adverse impacts they have from the species, and what are the control measures they adopt. A total of 50 people were interviewed. 

Satellite Data Analysis: To identify the factors influencing the detection of *Prosopis juliflora*, we analyzed datasets on weather conditions, land cover, and biophysical attributes. For weather data, we sourced monthly minimum, maximum, and diurnal temperatures from TerraClimate [22], along with average precipitation data during long rains, short rains, and dry periods from CHIRPS [23]. We examined soil organic carbon levels at various depths (0–5 cm, 5–15 cm, 15–30 cm, 30–60 cm) using global raster data from the World Soil Information Service (WoSIS) for biophysical variables [24]. We also incorporated slope and elevation data from specified sources and land cover classifications from the European Space Agency (ESA) [25,26]. Additionally, we analyzed monthly Normalized Difference Vegetation Index (NDVI) values for 2023 from Sentinel (10 m resolution) using the Google Earth Engine (GEE) [27]. Using the raster package in R Project Software version 4.1.0, we extracted data for each variable at the locations where Prosopis was present or absent. We provide detailed information on each dataset, including sources and resolutions, in Table 2. Given the varying spatial resolutions of these datasets, we adjusted our analysis for the landscape scale by clipping the datasets to our specific area of interest. We then resampled them to a standardized spatial resolution of 30 m using bilinear interpolation, which calculates the average value from the nearest four pixels (2 × 2 window) to the target pixel. We compiled this data into a raster stack, with each layer representing a different variable, enabling us to model the delineation of *Prosopis juliflora* from native vegetation across the study area.

### 3.3. Machine Learning Model

We investigated the efficacy of three machine learning algorithms—random forest (RF), support vector machine (SVM), and neural network (NN)—to pinpoint the variables that play a significant role in differentiating *Prosopis juliflora* from surrounding vegetation. To assess the importance of each variable, we conducted random forest regression where we regressed occurrence of species on a suite of variables (mentioned in Section 3.2) (Equtaion (1)). We measured the mean decrease in accuracy (% IncMSE) based on out-of-bag cross-validated predictions when each variable was randomly shuffled. Additionally, we also evaluated the predictive capabilities of each model by examining their overall accuracy, Cohen’s kappa coefficient, and the p-value from Mcnemar’s test. Overall accuracy is the ratio of the total number of correctly classifies pixels to the total number of reference pixels [28], the Cohen’s Kappa coefficient-the degree of similarity between the classification under analysis, and the random classification is measured by the Kappa value, with 0 representing identical classifications and 1 representing entirely distinct ones [29].

(1) $$\begin{array}{cc} Occurrenceof & Prosopis\;juliflora\sim \;\beta 0+\beta 1\;NDVI_{Feb}+\beta 2\;Max\;temp\\ & +\;\beta 3\;Min\;Temp+\beta 4\;Diurnal\;temp+\beta 5\;Avg\;ppt\;LR\\ & +\;\beta 6\;Avg\;ppt\;SR+\beta 7\;Avg\;ppt\;dry\;period+\beta 8\;LULC\\ & +\;\beta 9\;Elevation+\beta 10\;Slope+\beta 11\;NDVI_{Dec}\\ & +\;\beta 12\;SOC0-5\;cm+\beta 13\;SOC5-15\;cm\\ & +\;\beta 14\;SOC15-30\;cm+\beta 15\;SOC30-60\;cm+\varepsilon \end{array}$$

## 4. Discussion

The invasive spread of *Prosopis juliflora* in the rangelands of Africa presents a complex challenge that intersects ecological integrity and human livelihood. Our study highlights the application of artificial intelligence (AI) through machine learning (ML) algorithms in delineation of *Prosopis juliflora* from the native vegetation in Kenyan rangelands. Random forest algorithm performed best for classifying Prosopis and non-Prosopis regions, underscoring the profound capabilities of AI in navigating the complexities of ecological data, where the intricacies of environmental variability and species interactions present considerable analytical challenges [15,30,31]. This aligns with [32] assertion regarding RF’s adeptness at handling multidimensional and heterogeneous data, affirming its applicability in ecological studies where such characteristics are prevalent. However, all the three algorithms (RF, SVM and NN) are powerful and studies have shown that performance of these models can vary significantly depending on the nature of the dataset [33]. For instance, NN (deep learning models) require large amounts of data to effectively learn and generalize. In the scenario where data is limited or highly dimensional, NN might struggle to perform without overfitting. Since we used 80 data points in this study, NN did not perform well. Our study highlights insignificant response from NN model in absence of very large datasets. SVM require less data to perform but more data than RF to capture complex patterns effectively. RF on the other hand can handle high-dimensional data efficiently and is less prone to overfitting because of its bootstrapping and feature randomness approach making it well-suited for ecological datasets that are often noisy. Its ensemble approach disaggregates the decisions from the multiple decision trees, inherently handling both linear and non-linear relationships while on the other hand, SVM and NN require more tuning [32].

On analyzing the variables that contribute most to the identification *Prosopis juliflora* from the native background vegetation, we find that NDVI of February month, precipitation, landcover and elevation are instrumental in the detection of *Prosopis juliflora*. NDVI’s role as a significant indicator of vegetation health and dynamics, as highlighted by [34], underscores its indispensability in monitoring ecosystem changes. Previous studies also highlighted the importance of NDVI as an important variable if captured during dry season when the native vegetation dries up Prosopis continues to stay green due to its evergreen nature [15]. The predictive capability of NDVI is additionally reinforced by the observation that the presence and extent of Prosopis influenced greenness or NDVI levels, rather than NDVI being a determinant of its distribution. Precipitation, land cover and elevation were also found to be important variables that determine the delineation of Prosopis [30,31,35]. Temperature variables (max temp, min temp and diurnal temp) are not in the list of top six variables that contributes most to the delineation of Prosopis unlike some previous studies from Ethiopia [15,16].

Incorporating community insights into our analysis has unearthed a multifaceted perspective on the *Prosopis juliflora* invasion. The detrimental impacts on grazing lands and access routes articulate the profound challenges faced by communities, affecting their livelihoods and the ecological balance of local biodiversity. Conversely, the recognition of *Prosopis juliflora*’s benefits, such as contributions to soil stability and fuelwood supply, reflects the intricate nature of human-environment relationships [36]. Our study reveals that despite having a negative perception about *Prosopis juliflora*, pastoralists reported the species as the feed supplement, alternate source of protein for livestock [37]. Variations in the benefits obtained from *Prosopis juliflora* are evident across various professions, with activities such as beekeeping and participation in self-help groups (other occupation) reporting more benefits in contrast to farmers and pastoralists. These differences in benefit allocation among occupations may arise from the diverse resource needs and the ability of *Prosopis juliflora* to adapt to various livelihood strategies. For example, beekeepers might exploit the invasive species to their advantage by utilizing its flowers as a nectar source for bees. In contrast, farmers and pastoralists experience comparatively fewer benefits due to the difficulties in incorporating *Prosopis juliflora* into their farming or grazing routines. The noted benefit disparities could also be related to variations in financial returns and considerations of the environmental effects of this undesirable species on their way of life.

Notwithstanding its contributions, we acknowledge certain limitations in our study. The exploration of ML algorithms was somewhat constrained, hinting at the possibility of enhancing predictive performance and analytical efficiency through the incorporation of more variables. For example, we only considered data from one year (2023) but including data from multiple years might give us better insights and a more robust set of factors to study the classification of *Prosopis juliflora*. Additionally, the reliance on specific remote sensing data and indices (NDVI in this case), despite their utility, calls for careful consideration of potential limitations, including the risk of data misinterpretation under fluctuating environmental conditions. Addressing these limitations presents a clear trajectory for future research. Expanding the diversity of ML algorithms tested, integrating higher-resolution and temporal data, and exploring the incorporation of drone or UAV-based imagery could significantly augment the precision of *Prosopis juliflora* detection and mapping. Furthermore, conducting longitudinal studies would offer invaluable insights into the temporal evolution of *Prosopis juliflora* invasion, enabling the development of predictive model’s adept at forecasting future invasion patterns. Such models would be instrumental in informing proactive and adaptive management strategies, ensuring that interventions are both timely and tailored to the dynamic nature of invasive species proliferation. This forward-looking approach underscores the critical need for continuous innovation and interdisciplinary collaboration in tackling the challenges posed by *Prosopis juliflora* and similar invasive species, aiming for a sustainable balance between ecological integrity and community livelihoods.

## 5. Conclusions

Our study highlights the importance of integration of remote sensing, artificial intelligence and extension research, and adds unique dimension to our research. The use of AI for the detection of *Prosopis juliflora* in Kenya’s rangelands by showing the potential of ML algorithms, particularly the Random Forest model, to enhance the accuracy and efficiency of invasive species monitoring. The variables that emerged as key contributors that support delineation of *Prosopis juliflora* are NDVI (February month), precipitation, landcover and elevation. The identification of key environmental drivers contributing to *Prosopis juliflora* detection highlights the importance of integrating diverse data sources for a holistic understanding of invasion dynamics. We perceived mixed experiences from the local community highlighting both adverse and beneficial effcets of the invasive species. This duality calls for the adoption of holistic management strategies that judiciously consider both the ecological repercussions and the socio-economic welfare of the affected communities. Incorporating community perceptions into management strategies is a critical factor in ensuring that interventions are not only ecologically sound but also socially equitable and economically viable. This study contributes to the growing body of literature advocating for the integration of technological innovations into environmental management practices. As we advance, it is imperative that future research endeavors continue to refine these methodologies, explore the integration of additional data sources, and consider the complex socio-ecological systems within which invasive species management operates. Through such multidisciplinary approaches, we can better tackle the challenges posed by *Prosopis juliflora* and other invasive species, safeguarding ecosystem health, biodiversity, and the livelihoods of communities dependent on these critical rangeland ecosystems.

## Figures and Tables

**Table 1:** Comparison between different ML algorithms.

ML Classifiers	Overall Accuracy	Cohen’s Kappa Coefficient	Mcnemar’s Test *p*-Value
Decision Tree/Random Forest	95%	0.53	0.9
Support Vector Machine	78%	0.47	0.24
Neural Network	66%	0.12	0.07

**Table 2:** List of independent variables used in the study along with the data source.

	Variable	Description	Source	Spatial Resolution
1	Maximum Temperature (°C)	Maximum monthly temperature	[22]	4 km
2	Minimum Temperature (°C)	Minimum monthly temperature	[22]	4 km
3	Monthly Mean Diurnal Range (°C)	Difference between the maximum monthly temperature and minimum monthly temperature	[22]	4 km
4	Avg ppt for LR (mm)	Mean precipitation for long rain starts from March to May	[23]	4.8 km
5	Avg ppt for SR (mm)	Mean Precipitation starts from for short rain starts from October to December	[23]	4.8 km
6	Avg ppt for dry period (mm)	Mean Precipitation for dry season, which starts from June to September	[23]	4.8 km
7	Soil Organic Carbon (SOC) at 0–5 cm	Measure of Organic carbon density at layer 0–5 cm	[24]	250 m
8	Soil Organic Carbon (SOC) at 5–15 cm	Measure of Organic carbon density at layer 5–15 cm	[24]	250 m
9	Soil Organic Carbon (SOC) at 15–30 cm	Measure of Organic carbon density at layer 15–30 cm	[24]	250 m
10	Soil Organic Carbon (SOC) at 30–60 cm	Measure of Organic carbon density at layer 30–60 cm	[24]	250 m
11	Slope	Rate of change of elevation for each digital elevation model (DEM) cell.	[25]	30 m
12	Elevation	Digital Elevation Model (DEM)	[25]	30 m
13	LULC	Land cover types	[26]	10 m
14	NDVI	Vegetation Index	[27]	10 m

## Data Availability

The data presented in this study are available on request from the corresponding author.
